# CMD: a Cotton Microsatellite Database resource for *Gossypium *genomics

**DOI:** 10.1186/1471-2164-7-132

**Published:** 2006-05-31

**Authors:** Anna Blenda, Jodi Scheffler, Brian Scheffler, Michael Palmer, Jean-Marc Lacape, John Z Yu, Christopher Jesudurai, Sook Jung, Sriram Muthukumar, Preetham Yellambalase, Stephen Ficklin, Margaret Staton, Robert Eshelman, Mauricio Ulloa, Sukumar Saha, Ben Burr, Shaolin Liu, Tianzhen Zhang, Deqiu Fang, Alan Pepper, Siva Kumpatla, John Jacobs, Jeff Tomkins, Roy Cantrell, Dorrie Main

**Affiliations:** 1 Department of Genetics and Biochemistry, Clemson University, Biosystems Research Center, 51 New Cherry Street, Clemson, SC, 29634, USA; 2 ARS Crop Genetics & Production Research Unit, Stoneville, MS, USA; 3 ARS MSA Genomics Laboratory, Stoneville, MS, USA; 4 Clemson University Genomics Institute, Clemson University, Biosystems Research Center, 51 New Cherry Street, Clemson, SC, 29634, USA; 5 CIRAD, Centre International en Recherche Agronomique pour le Développement, 34398, Montpellier Cedex 5, France; 6 USDA-ARS, Southern Plains Agricultural Research Center, College Station, TX, 77845, USA; 7 USDA-ARS, WICS Research Unit, Cotton Enhancement Program, Shafter, CA, 93263, USA; 8 USDA-ARS, Crop Science Research Laboratory, P.O. Box 5367, Mississippi State, MS 39762, USA; 9 Biology Department, Brookhaven National Laboratory, Upton, NY 11973, USA; 10 Monsanto, St. Louis, MO 63167, USA; 11 National Key Laboratory of Crop Genetics & Germplasm Enhancement/Cotton Research Institute, Nanjing Agricultural University, Nanjing 210095, China; 12 Delta and Pine Land Company, Winterville, MS 38782, USA; 13 Dept of Biology, Texas A&M University, College Station, TX 77843, USA; 14 Dow AgroSciences LLC, Indianapolis, IN, 46268, USA; 15 Bayer BioScience N.V., Technologiepark 38, B-9052 Gent, Belgium; 16 Cotton Incorporated, Cary, NC, 27513, USA; 17 Department of Horticulture and Landscape Architecture, Washington State University, WA, 99164, USA

## Abstract

**Background:**

The Cotton Microsatellite Database (CMD)  is a curated and integrated web-based relational database providing centralized access to publicly available cotton microsatellites, an invaluable resource for basic and applied research in cotton breeding.

**Description:**

At present CMD contains publication, sequence, primer, mapping and homology data for nine major cotton microsatellite projects, collectively representing 5,484 microsatellites. In addition, CMD displays data for three of the microsatellite projects that have been screened against a panel of core germplasm. The standardized panel consists of 12 diverse genotypes including genetic standards, mapping parents, BAC donors, subgenome representatives, unique breeding lines, exotic introgression sources, and contemporary Upland cottons with significant acreage. A suite of online microsatellite data mining tools are accessible at CMD. These include an SSR server which identifies microsatellites, primers, open reading frames, and GC-content of uploaded sequences; BLAST and FASTA servers providing sequence similarity searches against the existing cotton SSR sequences and primers, a CAP3 server to assemble EST sequences into longer transcripts prior to mining for SSRs, and CMap, a viewer for comparing cotton SSR maps.

**Conclusion:**

The collection of publicly available cotton SSR markers in a centralized, readily accessible and curated web-enabled database provides a more efficient utilization of microsatellite resources and will help accelerate basic and applied research in molecular breeding and genetic mapping in *Gossypium *spp.

## Background

Comprehensive structural, functional and comparative studies of any genome are increasingly dependent upon the availability of an anchored physical map which shows the order of all genetic components in correspondence to their chromosomal localization. Anchoring of phenotypic information (such as trait or QTL) onto the physical map requires its integration with the genetic map of a genome which represents the relative positions of the genes and/or markers on chromosomes.

The International Cotton Genome Initiative (ICGI) was launched to facilitate the development of a saturated and fully integrated genetic and physical map of cotton [1]. A consensus linkage map is being developed by consolidating data generated by the cotton community using a common set of framework markers, such as microsatellites, or simple sequence repeats (SSRs) [2]. The generation of a transportable framework of SSR markers capable of being mapped in any segregating population was one of the major objectives of the ICGI. In keeping with the proposed goals of the ICGI, the Cotton Microsatellite Database (CMD) [3] has been initiated and funded by Cotton Incorporated. An Advisory Committee comprising both academic and industry representatives was formed to guide the development of CMD and to coordinate it with CottonDB [4], the genome database serving the international cotton research community.

Microsatellites consist of 1–6 repeating base pairs that are tandemly arranged in genomes [5]. While the number of repeats is highly polymorphic, the sequences flanking the repeats are highly conserved between individuals. The predominant mutation mechanism in microsatellite tracts is 'slipped strand mispairing', which generates the polymorphism with regard to the gain or loss of repeat motifs [6]. Microsatellites are abundant and widely distributed throughout the genomes of many higher plants and animals [7,8] and have been used extensively as molecular markers in the development of saturated linkage and physical maps [9-12].

Microsatellite markers are PCR-based, bi-parentally inherited, co-dominant markers. Polymerase chain reaction (PCR) products of different lengths can be amplified using unique primer pairs flanking the variable repeat microsatellite region after cloning and sequencing one allele. To develop microsatellite markers, primer sequences conserved between individuals and complementary to the microsatellite flanking sequences are identified by computer programs and synthesized.

SSR loci tend to be both multiallelic and highly polymorphic for repeat number, which is easily scored and used for genotyping. SSRs are amenable to analysis on automated DNA sequencers, and can thus be adapted to high-throughput genotyping. SSRs are often markers of choice due to their abundance, co-dominance, reproducibility, and ease of use [7,8]. As microsatellites are generally not as amenable to inter-generic studies as some other types of markers they are generally synthesized for each genus or species.

The applications of microsatellites for plant breeders are numerous. They can be used for gene tagging and genome mapping, for selecting progeny before a desired phenotypic trait is expressed, for localizing qualitatively as well as quantitatively inherited traits, improving the efficacy of selective breeding (particularly for traits with low heritability or that can only be measured in one sex), genetic diversity studies, variety protection, gene and QTL analysis, pedigree analysis, and for introgressing novel genes into breeding germplasm from exotic germplasm [8].

Microsatellites included in the CMD have been generated from several research groups within the international cotton community who are actively involved with generating, screening and mapping cotton markers. To make significant and timely advances in the genetic improvement of cotton, thousands of portable microsatellite markers are needed for the tetraploid genome of cultivated cottons. As these markers need to be characterized systematically prior to application, a standardized panel of 12 diverse genotypes was selected for screening from cultivated and exotic cottons [13]. This panel represents a balanced diversity of the core *Gossypium *germplasm (Table 1).

**Table 1:** A standardized panel of the cotton microsatellite marker database (CMD)

Panel Identity	Panelist	Description
CMD1	TM-1	*G. hirsutum *(AD_1_) genetic standard (BAC donor/RI parent)
CMD2	3–79	*G. barbadense *(AD_2_) genetic standard (fiber QTLs/RI parent)
CMD3	Acala Maxxa	California Upland cotton (AD_1_) and BAC donor
CMD4	DPL 458BR	Upland cotton (AD_1_) with significant acreage
CMD5	Paymaster 1218BR	Upland cotton (AD_1_) with significant acreage
CMD6	Fibermax 832	Upland cotton (AD_1_) with significant acreage
CMD7	Stoneville 4892BR	Upland cotton (AD_1_) with significant acreage
CMD8	Pima S-6	Pima (AD_2_) germplasm breeding source
CMD9	*G. arboreum *(A2-8)	A subgenome representative
CMD10	*G. raimondii *(D5-3)	D subgenome representative
CMD11	*G. tomentosum *(AD_3_)	Introgression breeding source
CMD12	*G. mustelinum *(AD_4_)	Introgression breeding source

Note: Cotton plants and DNA stocks are maintained by Dr. John Yu at USDA-ARS, College Station, Texas.

The major goals of the CMD are:

(1) to collect and integrate all the publicly available cotton microsatellite data in a centralized, curated, non-redundant online oracle database,

(2) to provide access to the CMD standardized panel screened data,

(3) to provide a set of comprehensive interface tools for rapid data retrieval,

(4) to provide a suite of stand-alone microsatellite data mining tools,

(5) to provide a communication portal for collaboration within the cotton research community.

## Construction and content

### Database and web interface development

Currently, the database is composed of 14 tables which store all the data for the microsatellite projects including information on project collaborators, SSR-containing clones, sequences, primers flanking the SSRs, repeat motif, open reading frame position, genetic markers and maps, standardized panel varieties, and data homology, and publications. In a separate but linked database within CMD, the CMap schema consists of 16 tables including information about genetically mapped cotton SSRs. Data for cotton SSR markers and genetic maps, as well as panel screened cotton microsatellites, are submitted by researchers and then curated for any potential errors prior to uploading to the database using scripts written in Perl version 5.8.2. Web interfaces for database query and the query result pages are also developed in Perl.

### CMD microsatellite data projects

In cotton, the first SSR markers were developed at the Brookhaven National Laboratory (prefix "BNL"). The 379 BNL microsatellites presented through CMD were derived from *G. hirsutum *small insert genomic library enriched for (GA/CT)n and (CA/GT)n inserts [14]. Later, the 309 JESPR [15] and 392 CIR [16] microsatellites were developed by streptavidin capture of 5'-biotinylated microsatellite-enriched libraries. The 53 CM microsatellites were developed using randomly sheared (nebulized) genomic DNA for adapter-ligation, rigorous removal of biotinylated oligos, and high-density colony blots for constructing enriched libraries [17]. The 84 MGHES [18], 1169 MUSS/MUCS [19] and 1032 NAU [20,21] microsatellites were developed by screening public databases for EST-derived SSRs. The 750 TMB [22] (Yu et al., 2002) and 1316 MUSB [23] cotton microsatellites are BAC-derived.

The individual project pages contain access to all public data currently available for each microsatellite project, all of which have been approved by the project principal investigator. The standardized project information includes: a project summary abstract, investigator contact information, related publications, microsatellite information, including GenBank accession numbers, clone sequences, primer sequences, repeat motif, standardized panel screened data (if available), mapping data, and any homology with known proteins. Marker data, primers, microsatellite sequences and standardized panel screened data are available for download directly from each project page as well as an overall downloads page. Currently, CMD contains information on 5,484 annotated cotton microsatellites which can be viewed and downloaded. Annotation of the sequences is periodically updated so that our data reflects changes in protein records in the NCBI GenBank non-redundant protein database.

A microsatellite information page displays the sequence along with the repeat sequence and primers. The longest putative open reading frame (ORF) is also marked in color in the sequence along with the microsatellites. SSRs in the non-coding region tend to be more polymorphic and those in the coding region tend to be more transferable among species so the information of SSR position in a gene structure will be useful for marker development [24].

Currently, 3,452 of the cotton microsatellites available through CMD have been checked for internal redundancy. Any of the following criteria were considered as redundant: 1) identical GenBank accession number; 2) completely identical primer pairs; 3) identical forward primers; 4) identical reverse primers; 5) forward primer identical to reverse and vice versa. From this analysis, 3,135 (90.8%) of the microsatellites checked were considered to be unique and were noted accordingly in the database.

### A standardized panel of *Gossypium *genotypes for systematic characterization of cotton microsatellite markers

Upon extensive discussion and consultation, a standardized panel of 12 *Gossypium *genotypes for cotton microsatellite database (CMD) was established [13,25]. This genotype panel represents a balanced diversity of the core *Gossypium *germplasm including cultivated and exotic cottons as shown in Table 1. Among the CMD standardized panel representative genotypes, TM-1 and 3–79 are the genetic standards for AD_1 _(G*. hirsutum*) and AD_2 _(*G. barbadense*) species, respectively. Because TM-1 and 3–79 are also parents of a permanent RIL mapping population, they are essential for the integrated genome mapping and selection of the core reference markers. Acala Maxxa is California Upland cotton from which a BAC library is also constructed. DPL 458BR, Paymaster 1218BR, Fibermax 832, and Stoneville 4892BR are Upland cotton representatives with a significant acreage across the Cotton Belt and beyond. These Upland selections represent the contemporary Upland cotton variability and extend the Upland cotton diversity. They are often used as the National Variety Test (NVT) standards that provide a database of agronomic performance for any agronomic comparisons. Pima S-6 is the source of *G. barbadense *Pima germplasm breeding programs. *G. arboreum *(A_2_) and *G. raimondii *(D_5_) are representatives of A and D subgenomes, respectively. *G. tomentosum *(AD_3_) and *G. mustelinum *(AD_4_) are possible sources of introgression breeding programs (Table 1).

For each of 12 cotton genotypes three to five individual plants are maintained in a USDA-ARS greenhouse in College Station, Texas [25]. For each genotype only one single plant is flagged for tissue harvest and DNA extraction. The standardization of panel DNA stocks provides the best uniformity for cotton researchers with ongoing SSR marker development. Polymorphisms arising from easily assayed variation in SSR numbers show great utility in crop genetic mapping and other applications. With this standardized genotype panel, cotton SSR markers derived from different sources or groups can be evaluated in a systematic way to minimize the potential redundancy and to determine the markers' Polymorphic Information Content (PIC) values for ready applications. In addition to the information on the clones, sequences, primers, amplification conditions and fluorescent primer labels used, the amplified fragments sizes are currently available in CMD for the 375 BNL, 204 CIR, and 127 JESPR microsatellites screened against the standardized panel. The timetable for the inclusion of further panel screened data is also available through the CMD.

### Genetically anchored mapping data

A genetically anchored physical map for cotton is being developed using cotton BAC libraries [2]. Through various genetic markers, including SSRs, the cotton physical map will be anchored on the future consensus cotton genetic map [2]. CMD stores and presents currently available data for major cotton genetic maps with mapped SSRs that were constructed for different crosses. Currently, CMD contains data for four genetic maps: 1 - BC_1_: ((Guazuncho2 (*G. hirsutum*) × VH8-4602 (*G. barbadense*)) × Guazuncho2) [16,26]; 2 - F_2_: *G. hirsutum *race 'Palmeri" × *G. barbadense *Acc. "K101" [11]; 3 - BC_1_: (TM-1(*G. hirsutum*) × Hai7124 (*G. barbadense*)) × TM-1) [20,21]; 4 - RIL: TM-1 (*G. hirsutum*) × 3–79 (*G. barbadense*) [19]. The anchored genetic markers can be viewed in several formats, including an excel spreadsheet, a database search interface, and a graphical interface for comparative visualization of SSR maps. Additional data on the newly mapped SSRs will be available soon.

## Utility and discussion

### Database access

**Figure 1:**
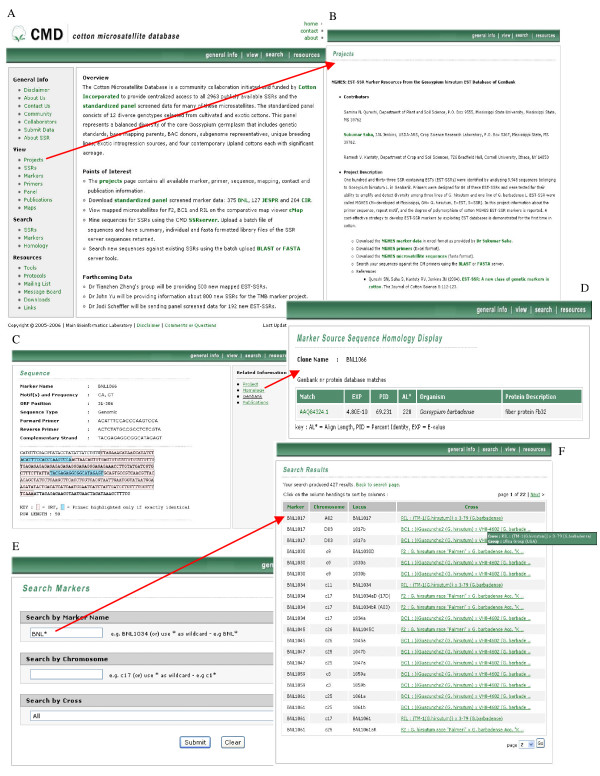
**CMD home page and the representative search pages**. This figure illustrates the CMD homepage (A) and the representative search pages; microsatellite projects (B), marker search and view, homology search (C-F).

The CMD website is composed of general information pages (Figure 1A), including CMD tutorials, project pages (Figure 1B), database query/browse interfaces and other tools such as a comparative map viewer CMap, sequence similarity server, SSR server, and CAP3 server. The CMD web pages are organized such that users can easily access the data of interest regardless of the navigation starting point. For example, the microsatellite project pages (Figure 1B) have links to the CMD standardized panel pages, marker detail pages, sequence files in FASTA format, a downloads page, sequence similarity server, or abstracts for the related publications. Similarly, the CMD standardized panel (Table 1) details page has links to the SSR project detail page. A general CMD tool bar is also included in each page to aid the ease of navigation through the site.

#### Database search interface

The initial SSR search result page displays SSR identifiers. The individual SSR entry links to a page where details of the SSR are displayed (Figure 1C) with links to the corresponding project page, the top protein homolog identified through a sequence similarity search (Figure 1D), microsatellite sequence in GenBank, and related publications. Markers can be searched by marker name, chromosome, or cross (Figure 1E,F). Other pages include a mailing group list form, so users can exchange information and be kept up to date on new developments in CMD. The message boards automatically list all the information exchanged by the mailing list. The links page contains appropriate cotton links.

#### Graphical interface to maps

**Figure 2:**
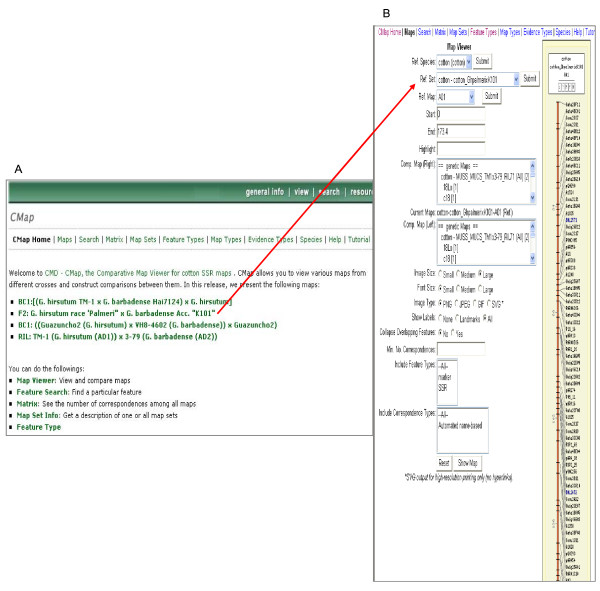
**Cotton CMap Viewer**. A. The opening page of CMap Viewer with 4 major genetic maps of cotton. B. An example of individual linkage group pages with the associated markers.

CMD also provides a graphical tool CMap in which the cotton genetic maps (Figure 2A) are displayed with the number of anchored SSR markers (Figure 2B), and the location of mapped SSRs is compared between different crosses of cotton. CMap is part of the Generic Model Organism Database [27]. CMap allows the user to select the map of interest and the maps for comparisons. The feature search looks for a certain feature by name or accession ID, species, and feature type. The CMap correspondence matrix allows users to view the number of correspondences among all selected maps.

### Analysis tools

The CMD tools page provides access to an SSR server, a CAP3 Assembly server, and a sequence similarity server that includes BLAST and FASTA search tools.

#### SSR server

SSR analysis is performed using a modified version (SSR) of a Perl script SSRIT [28] with parameters set to detect mono- to hexanucleotides of user specified length. To examine the location of SSRs in the sequences in relation to the putative coding region, the SSR server uses the FLIP [29] program which is available through the Organelle Genome Megasequencing Project [30]. FLIP is a UNIX C program that finds/translates ORFs (open reading frames) in sequences. Using the FLIP output, the longest ORF is identified and the relative SSR location is reported. Potential primers are identified using Primer3 [31].

Using the SSR server, users can upload a batch of sequences in FASTA format and select the motif type and repeat length to search. After job completion, users are redirected by email to a web page providing 1) a summary report of the SSR analysis, 2) a library file of the uploaded sequences, 3) a library file of the SSR containing sequences, and 4) an excel file of the individual properties of the SSR-containing clones. The individual properties include sequence name, length of the SSR-containing sequence, repeat(s) motif and number, SSR start/stop position, ORF start/stop position, primer pairs, SSR location relative to the ORF, and GC content of the sequence.

#### CAP3 server

To reduce the inherent redundancy and increase transcript length ESTs are routinely assembled into longer consensus sequences, also known as contigs. We have implemented the contig assembly program CAP3 [32] as an online server to allow users to assemble ESTs prior to mining the consensus sequences for microsatellites using the CMD SSR server. Users can upload quality files for their sequences and specify the percentage identity in the overlap region (p value). While the quality and quantity of EST data varies greatly for each species, we have found that using a high level of stringency (p = 90 or 95) tends to prevent over assembly and helps distinguish between gene family members. Assembling ESTs that come from the same transcript is a common method of creating a putative unigene for an organism. As more ESTs are sequenced and added to the public domain, the cotton unigene can be continually refined using the CAP3 server and mined for SSRs using the SSR server.

#### Sequence similarity servers

The online BLAST and FASTA sequence similarity search servers allow users to perform homology searches between their sequences of interest and the annotated SSR sequences and primers in CMD. From the web interface, researchers can upload a file of sequences, select the search algorithm (e.g. BLAST, FASTA), the database (SSR sequences, SSR primers) and submit their job for processing. Once the job has completed an email is sent with a URL providing secure access to the results of the search. From the URL, users retrieve a summary of the search with the number of sequences that had matches with the database selected, an excel file containing the best match, any known function, match organism, match length, percent identity, expectation value, alignment length, and start and stop alignment positions. Our sequence similarity server, specifically designed for CMD researchers, will help users compare new sequences and primers against existing microsatellites and help decrease redundancy of effort in developing new markers. As we migrate the sequence similarity servers to a computational cluster, we plan to add the following databases: NCBI cotton ESTs, TIGR cotton gene indices, NCBI cotton genomic sequences and NCBI cotton protein sequences.

### Future development

Future development will focus on the establishment of a standard nomenclature of cotton SSRs, adding new microsatellite data, improving the tools and functionality of the web interface, such as an advanced search site with options for search/display categories, full sequence processing facilities for cotton researchers, and a quarterly newsletter for the cotton community. The annotation of the SSRs with known homology will include further classification using the gene ontology terms associated with the matching sequences in the Swissprot database. When the physical map is available, users also will be able to retrieve the anchored BAC clones containing the SSRs of interest through the anchored BACs page in the map viewer. Data that are currently scheduled to be added in the near future include 800 BAC-derived cotton genomic SSRs and 500 cotton EST-SSRs from the public domain, and 200 SSRs from private companies.

## Conclusion

The CMD has been initiated to provide researchers, engaged worldwide in cotton research, with centralized access to microsatellite markers, an invaluable resource for basic and applied research in cotton breeding. As such, the CMD serves the cotton community as a major repository of the publicly available cotton microsatellite data and a unique repository for the CMD standardized panel screened data, a key tool for systematic characterization of the SSR markers developed for cotton. Access to this data is provided through integrated web tools which allow users to directly access individual or combined project data via search interfaces which provide download and visualization of microsatellites, their flanking primers, open reading frames (ORFs), and SSR genetic maps. CMD also provides a suite of online tools for data analysis of new and existing microsatellites through its SSR, CAP3, and FASTA/BLAST servers. Overall, the CMD serves as a major resource for the international cotton community, and can be viewed as an important vehicle toward increased collaboration among cotton scientists.

## Availability and requirements

CMD is publicly available at the URL . The CMD is a relational database implemented using the Oracle Relational Database Management System version 9.2.0. Users can subscribe to the CMD mailing list, but registration is not required to use the CMD.

## Abbreviations

BAC – Bacterial Artificial Chromosome

BC1 – Backcross 1^st ^generation

BLAST – Basic Local Alignment Search Tool

CAP3 – Contig Assembly Program

CIRAD – Centre International en Recherche Agronomique pour le Développement

CIR – CIRad

CM – Cotton Microsatellites

CMap – Comparative Map

EST – Expressed Sequence Tag

EXP – Expectation Value

FASTA – Fast All alignment search tool

FLIP – FLexible In-system Programmer

JESPR – Jenkins, El-Zik, Saha, Pepper, Reddy microsatellite repeats

MGHES – Mississippi *Gossypium hirsutum *EST-SSR

MUCS – Microsatellite Ulloa Complex Sequence repeats

MUSB – Microsatellite Ulloa Simple BAC repeats

MUSS – Microsatellite Ulloa Simple Sequence repeats

NAU – Nanjing Agricultural University

QTL – Quantitative Trait Loci

RIL – Recombinant Inbred Line

TMB – TM-1 genetic standard BAC/BIBAC libraries microsatellite repeats

## Authors' contributions

AB participated in the database and interface design, performed general CMD data collection, organization and curation. CJ, SM, PY participated in the database and interface design and construction, and developed scripts for database upload and sequence processing. SJ implemented CMap, uploaded mapping data and was involved with the database schema design and implementation, SF managed the oracle system and was involved in database schema design and tool development, MS performed all homology searches and was involved with database design and construction, RE helped design and implement the SSR, FASTA, BLAST and CAP3 servers, JS and BS screened the cotton SSRs against the CMD standardized panel. MP participated in the development of the MUSB microsatellite project and participated in the database and interface design. JML developed and provided data for the CIR project and participated in the CMD general data collection and organization. JY established, maintained, and distributed the 12-genotype CMD panel DNA stocks as well as developed and provided data for the TMB project and participated in the general coordination of the project. MU developed and provided data for the MUSS/MUCS and MUSB projects, participated in collection and organization of mapped SSR data presented in the CMD. SS was the lead scientist in developing MGHES and provided data for the MGHES and JESPR projects and was involved in the general coordination of the project. BB and SL developed and provided data for the BNL project, critically reviewed and contributed to database and manuscript development. TZ developed and provided data for the NAU project, critically reviewed and contributed to database and manuscript development. AP participated in the development of the JESPR project, critically reviewed and contributed to database and manuscript development. DF performed redundancy analysis of the CMD microsatellites and was involved in the general coordination of the project. JT participated in the development of the MUSB microsatellite project. SK and JJ served on the CMD Advisory Board, critically reviewed and contributed to database and manuscript development. RC conceived and performed general coordination of the project. DM supervised the project and was involved with all aspects of database design, construction and implementation. All authors read and approved the final manuscript.
